# The power of social connection and support in improving health: lessons from social support interventions with childbearing women

**DOI:** 10.1186/1471-2458-11-S5-S4

**Published:** 2011-11-25

**Authors:** Rhonda Small, Angela J Taft, Stephanie J Brown

**Affiliations:** 1 Mother and Child Health Research, La Trobe University, 215 Franklin Street, Melbourne Victoria 3000, Australia; 2 Healthy Mothers Healthy Families Research Group, Murdoch Childrens Research Institute, Flemington Road, Parkville Victoria 3052, Australia

## Abstract

**Background and objective:**

Social support interventions have a somewhat chequered history. Despite evidence that social connection is associated with good health, efforts to implement interventions designed to increase social support have produced mixed results. The aim of this paper is to reflect on the relationship between social connectedness and good health, by examining social support interventions with mothers of young children and analysing how support was conceptualised, enacted and valued, in order to advance what we know about providing support to improve health.

**Context and approach:**

First, we provide a brief recent history of social support interventions for mothers with young children and we critically examine what was intended by ‘social support’, who provided it and for which groups of mothers, how support was enacted and what was valued by women. Second, we examine the challenges and promise of lay social support approaches focused explicitly on companionship, and draw on experiences in two cluster randomised trials which aimed to improve the wellbeing of mothers. One trial involved a universal approach, providing befriending opportunities for all mothers in the first year after birth, and the other a targeted approach offering support from a ‘mentor mother’ to childbearing women experiencing intimate partner violence.

**Results:**

Interventions providing social support to mothers have most often been directed to women seen as disadvantaged, or ‘at risk’. They have also most often been enacted by health professionals and have included strong elements of health education and/or information, almost always with a focus on improving parenting skills for better child health outcomes. Fewer have involved non-professional ‘supporters’, and only some have aimed explicitly to provide companionship or a listening ear, despite these aspects being what mothers receiving support have said they valued most. Our trial experiences have demonstrated that non-professional support interventions raise myriad challenges. These include achieving adequate reach in a universal approach, identification of those in need of support in any targeted approach; how much training and support to offer befrienders/mentors without ‘professionalising’ the support provided; questions about the length of time support is offered, how ‘closure’ is managed and whether interventions impact on social connectedness into the future. In our two trials what women described as helpful was not feeling so alone, being understood, not being judged, and feeling an increased sense of their own worth.

**Conclusion and implications:**

Examination of how social support has been conceptualised and enacted in interventions to date can be instructive in refining our thinking about the directions to be taken in future research. Despite implementation challenges, further development and evaluation of non-professional models of providing support to improve health is warranted.

## Introduction

Everybody ‘knows’ that when people experience good social support and feel connected to family, friends and community, their health is better. What is much less clear is how to achieve this sense of connectedness for those who feel unsupported. Some people are more likely to experience social isolation, and/or social exclusion, linked often to the complexity of their daily lives and poorer health and wellbeing: women experiencing violence, newly-arrived immigrants, mothers of young children, Indigenous Australians, people experiencing depression or other stigmatising illnesses like HIV, to name just a few.

One possible public health response is to attempt to provide the social support that appears lacking, in an effort to improve general wellbeing and health. Is this feasible, or indeed desirable? How can social support be enacted: what does such support mean in the context of public health interventions? And what are the challenges and pitfalls?

In this paper we attempt to address these questions drawing on what has been done to provide support in the early years of motherhood, as indeed this has been a common focus for public health ‘support’. First, we provide a brief recent history of the types of social support provided for mothers with young children. We critically examine what was intended by ‘social support’, who provided it and for which groups of women, how support was enacted and what was valued by women. Second, we examine the promise and challenge of lay (sometimes termed peer, para-professional or non-professional) social support approaches focused explicitly on companionship, and draw on experiences in two cluster randomised trials which aimed to improve the wellbeing of mothers. One trial involved a universal approach, providing ‘befriending’ opportunities for all mothers in the first year after birth, and the other a targeted approach offering support to childbearing women experiencing intimate partner violence from a local ‘mentor mother’. Finally, we reflect on the lessons learned from these two trials for informing future research on social support interventions to improve health.

## Support for mothers with young children: a brief overview

Across the developed world various public health interventions have been implemented to provide support to women in the early years of motherhood. Most have involved support given by primary health care professionals, almost always nurses. Some interventions have been universal, offering healthcare and support to all women after giving birth. One example is the home visiting services provided by community midwives and health visitors in the first weeks after childbirth in the UK. Another is the local network of Maternal and Child Health Nurse (MCHN) centres established in the state of Victoria, Australia in the early 1920s, initially as a targeted welfare program for socially disadvantaged mothers but now providing a universal health, information and advice service to families in the early childhood years.

The interventions targeted to particular groups of women - most typically young, single or impoverished women - have generally been directed to those women whose children are seen to be ‘at risk’ of poor birth outcomes, including preterm birth and low birth weight [1], poorer developmental outcomes, or child abuse and neglect. For example, targeted nurse home-visiting programs burgeoned in the US in the 1980s [^2^] and were followed a decade later by similar programs in the UK and Australia, despite much stronger primary care support services for recent mothers already in existence in both these countries. In the state of Victoria, Australia, an enhanced home visiting service developed within the universal MCHN service in the 1990s, providing additional support to families identified as ‘vulnerable’ in some way, including: families with drug and alcohol, mental health or family violence issues; families known to child protection services; families experiencing homelessness; unsupported parents under 24 years of age; low income, socially isolated, single parent families; families with significant parent/baby bonding and attachment issues; parents with an intellectual disability; children with a physical or intellectual disability; and infants at increased medical risk due to prematurity, low birth weight, drug dependency or failure to thrive [3].

Some of these types of programs are initiated during pregnancy and offer home visiting for up to two years after the birth. Others have simply offered support postnatally. Although less common, there are also examples of lay support programs established for vulnerable families, such as the Resource Mother Program in the US [4], the Community Mothers’ Programme in Ireland [^5^] and Newpin – the New Parent and Infant Network – in the UK [6]. The effectiveness of such nurse and lay home visiting programs for disadvantaged mothers remains somewhat unclear, due in part to the diversity of programs implemented and populations targeted, their varied outcomes of interest, and lack of rigorous evaluation. We return to this in the Discussion.

Broadly speaking what all these programs have in common is a focus on healthy child development and support for parenting. That is, they have a strong information, advice and education purpose to support (and improve) women’s parenting skills. The primary objective is to improve child outcomes. Most also have some focus on enhancing women’s self-confidence in parenting, and some aim to raise self esteem among women experiencing adversity of various kinds (violence, poverty, depression). Few, if any, have aimed simply to provide companionship or friendship or to focus primarily on improving maternal outcomes. What is also notable is the dearth of postnatal support programs offered to new parents as couples. One recent exception aimed to address maternal fatigue and improve maternal mental health (anxiety and depression) via a universal psycho-educational intervention delivered to couples by experienced maternal and child health nurses, with a focus on improving the quality of the intimate partner relationship after birth and enhancing infant management [7].

Labour and birth have also been the focus of support interventions for mothers. Here childbirth education classes are perhaps the most common type of support offered to pregnant women leading up to birth and this approach is very focused on preparation and education for labour and birth, with little high quality evidence of effectiveness to date [8]. There have also been efforts in recent decades to ensure continuous support in labour. Some involve support from a midwife, but others focus on partners or other non-health professional support people (or ‘doulas’). Such programs have aimed to achieve more empowering, satisfying and supported birth experiences for women, reducing the need for medical interventions of various kinds. Here the motivation for supporting women is to increase women’s confidence in their capacity to birth, to encourage, praise and empower women by providing companionship and reassurance, as well as physical contact and comfort [9,10]. Education or information-giving mostly plays little or no role in these labour support interventions.

Further support provision to mothers has occurred in the voluntary or community sector, with the activities of a range of organisations, such as those providing breastfeeding support (eg Australian Breastfeeding Association (ABA), La Leche League International), or support for women who have multiple births, for parents of children with disabilities or for those experiencing mental health problems such as depression. Mothers’ groups and playgroups for women with young children also exist in various formats in many communities providing opportunities for mothers to meet and share experiences of life with young children. Some of these support activities are facilitated by health professionals, but many involve women meeting and supporting each other very informally, or sometimes with systems of training in place for volunteers to provide specific kinds of support (as for example in ABA with breastfeeding support and advice). For the most part, what these activities aim to provide for women is a sense that they are not alone – with breastfeeding issues, feeling overwhelmed or down, having twins, etc – that there are indeed other mothers to talk to, and share experiences and information with. These activities are most often based on mutual support, reciprocity and friendship.

Telephone-based support provided by peers has also been offered. In Australia, for example, Panda (Post and Antenatal Depression Association) is an organisation that has provided a telephone helpline for mothers experiencing depression. Trained and supported ‘peers’ who have also experienced depression, speak with women over the phone, in a system also integrated with back-up professional counselling for those more severely depressed. (http://www.panda.org.au) Telephone-based peer support for mothers has been evaluated in two Canadian trials, both showing peer support by telephone to be effective – improving breastfeeding rates in one trial [11] and reducing maternal depression in the other [12]. And a systematic review of 14 trials of pro-active telephone-based peer support has indicated evidence of effectiveness also in preventing smoking relapse and possibly low birth weight [13].

In recent years there has also been an explosion of web-based support networks and programs for mothers. These are diverse in focus and intent, with some designed to inform or to educate, and others simply to connect women to share their experiences of motherhood or find out about activities available for mothers. One of many possible examples is Netmums in the UK, which describes itself as: “…a family of local sites that cover the UK, each site offering information to mothers on everything from where to find playgroups and how to eat healthily, to where to meet other mothers.” (http://www.netmums.com). As yet, evaluation of the impact of such internet networks is scant, and we mention them here merely to acknowledge the existence of this newer form of support activity.

Clearly the meaning of social support, who provides it and the motivation for offering it to women at the time of childbirth and early mothering varies considerably, as seen in the range of examples briefly canvassed here. Support strategies for mothers have been subjected to evaluations of varying quality, with mixed findings of effectiveness overall. And there has been little evaluation comparing types of support provided or comparing the effectiveness of different providers of support (i.e. lay vs. professional support, or nurse vs. other types of professional support).

It is interesting – and important – to consider what women say they value in receiving support, and to compare this with what support is offered. Such information is infrequently collected or available, but when it is, the things women appear to value about receiving support are non-judgemental companionship or a listening ear, reassurance and feeling less alone [14-17]. The educational and informational benefits of the support provided are less emphasised by women, despite the clear focus on these aspects in most social support interventions. Feeling isolated and alone are common experiences for women in the early months of motherhood, so it is unsurprising that women value the offer of reassurance and companionship so highly.

We have been involved in two randomised trials of pragmatic public health intervention strategies involving the offer of social support to recent mothers. In both studies social support was conceptualised and implemented, not in terms of information or education for women, but rather as befriending and companionship. The primary aim of both interventions was to improve maternal wellbeing, both emotional and physical, rather than to improve child health outcomes; although we did hypothesise that there would be flow on benefits for children’s health resulting from improvements in women’s health. In the next section we outline some of the issues and challenges inherent in this approach to providing support.

## Enacting social support as social connection for mothers

### Introducing PRISM: why befriending?

PRISM – Program of Resources, Information and Support for Mothers – was a community intervention trial evaluating primary care and community-based strategies for improving the health of mothers in the year after childbirth. It was conducted in 16 metropolitan and rural municipalities in Victoria, Australia over a two year period from late 1998 to 2000. PRISM drew on social ecological theory with program development building on existing services and capacities of local communities. Intervention strategies aimed to improve women’s health after birth by increasing knowledge of common postnatal problems, increasing the availability and accessibility of ‘someone to talk to’, and developing social networks appropriate to each community. Mother-to-mother support based on the principle of non-professional befriending was one of four key elements in the intervention protocol [18]. Other key elements were: training for primary care practitioners (maternal and child health nurses, general practitioners) to increase recognition and responsiveness to maternal physical and psychological health problems, an information kit including information about local services, establishment of a local steering committee to oversee and tailor strategies in each community, and a community development officer based in each community for two years to support program implementation and sustainability [18].

Befriending strategies in PRISM were about communities finding ways to increase the chances that women with young babies would make friends with other mothers in their own local community. The decision to focus on mother-to-mother support was informed by in-depth interviews with 90 women, half of whom had depressive symptoms around 8-9 months after having a baby. Isolation, physical health issues, and partners who were absent for more than 10 hours a day, five or more days a week were common problems faced by new mothers in the year after childbirth. Many women who had been depressed talked about putting on a ‘brave face’ whenever they went out, and only going to see the maternal and child health nurse when they were having a ‘good day’. First time mothers’ groups – offered by most maternal and child health services in Victoria – were highly valued by many mothers, but others said they didn’t go because their baby was too unsettled, they didn’t like groups, or they worried about being judged by other mothers or by their maternal and child health nurse. Historically, mothers’ groups offered by maternal and child health services have mainly targeted first-time mothers. Listening to first-time and other mothers talk about their isolation and concerns about being judged, we were struck by how often women put on a brave face to the world while simultaneously struggling with the realities of caring for a newborn infant 24 hours a day, seven days a week, often with very little ‘time out’. Women had invited us into their homes, and shared very personal stories. They often commented on how much they enjoyed talking with us. Our decision to incorporate mother-to-mother befriending strategies in PRISM grew out of the conversations we had with women in this formative research that was subsequently published in a book called Missing Voices: the experience of motherhood [19].

Befriending in PRISM was about local communities finding ways to increase the chances that women with young babies would make friends. It was conceived and implemented as a universal strategy intended to provide mother-to-mother friendship and support. It was not about improving parenting skills, educating mothers, encouraging help-seeking or preventing child abuse. We all need friends. Friendships develop for all sorts of reasons. Chance often plays a big role, and friendships cannot be readily ‘engineered’ by others, which is perhaps why befriending was one of the most challenging strategies to implement in PRISM.

In the first year of PRISM, befriending initiatives developed slowly as steering committees, maternal and child health nurses, community development officers (CDOs) and the research team grappled with how universal, rather than targeted, opportunities for befriending could most appropriately be provided. Our aim was to implement a range of befriending strategies in local communities promoting time out for mothers and the importance of mothers looking after their own health and wellbeing. Local mothers were consulted, both through steering committees, specially organised meetings with mothers, and via small local surveys. Ideas were tried out; some gained traction, while others did not. A variety of small-scale, largely informal befriending opportunities emerged. Connecting mothers so they could enjoy something together (an activity, time, relaxation) was a common strategy, and included identifying and ‘naming’ mother-friendly places where women could meet (eg cafes, community venues), setting up activities for mothers (eg pram walking times, Mothers’ Day lunches), and making connections between mothers though a facilitator (eg the maternal and child health nurse).

So befriending in PRISM developed as a range of strategies, rather than being a single program. Invariably, lots of ideas were discussed by local Steering Committees. Some were implemented and maintained. Lots of sharing of ideas between areas occurred, particularly as a result of contact between the CDOs. Befriending initiatives were facilitated by a whole range of people and organisations, including: PRISM community development officers, maternal and child health nurses, staff in community houses, libraries, community health services and local businesses such as cafes, cinemas and leisure centres. See Box 1 for a more detailed account of the range of befriending opportunities that developed.

By midway into the second year of PRISM implementation, befriending had become highly valued by community stakeholders as a key element of the project. The findings of a Communities’ Feedback Survey sent to steering committees, mothers, primary care and community agencies in each area, demonstrated that 90% of stakeholders thought that provision of ‘increased opportunities for mothers to meet and do things they enjoy with other mothers’ had been achieved in their area, and over 80% of mothers and maternal and child health nurses surveyed thought it was important that such befriending opportunities for mothers be maintained.

Feedback from women taking part in befriending activities also indicated that they valued these opportunities to meet other mothers:

*‘When Sean was born I spent a few hours every day taking him for a walk in the park. I was home with no car so walking was my only way to get around. On a visit to the health centre I saw a flyer about mums walking together for fun. As I walked everywhere anyway I thought it would be nice to walk with others mums. Months down the track we all still walk together. Everyone has their own reasons for coming*, *for most of us it is for the exercise. One of the mothers used to come with her four week old but then had to return to work. Because of this she missed out on joining a mums’ group and couldn’t come walking. She has since changed her days at work just to come walking*, *as she is finding herself desperate for some other mums to talk to*, *who can relate to what she is talking about.’*

*‘Personally*, *being involved in PRISM … has meant an expansion of my friendships and networks in the area*, *which prior to this were limited*, *as pre-motherhood I did not work in the area and I had only lived [here] for some six months before my baby was born.’*

‘Coming to the monthly Mothers’ Lunches meant that I met a mother who lives a few streets away. We’ve both got babies about the same age and we’ve now become firm friends.’

Nevertheless, and despite very little evidence of similar activity occurring in comparison areas, there was no evidence in women’s responses to the trial outcome survey that befriending strategies had substantially increased the likelihood of women in the intervention communities establishing new friendships, nor had they reduced women’s sense of social isolation. Nor did the range of strategies implemented in PRISM – of which befriending was one – influence the prevalence of depression for mothers in intervention communities compared with those in comparison areas [20]. Box 2 provides a brief summary and references for the key features and findings of PRISM.

### Introducing MOSAIC: why ‘mentoring’?

*‘The best thing was that I had* someone *to talk to about things*, *who was there for me and cared about how I was feeling… it was beneficial. The visits and phone calls and texts have been beneficial. You work up a friendship basis*, *going to lunch and shopping and stuff. If the mentor hadn’t come here*, *I wouldn’t have gone out of the house.’*

In contrast, MOSAIC (MOtherS’ Advocates In the Community), was a pragmatic trial of targeted social support that aimed to reduce intimate partner violence and depression by providing up to twelve months’ home visiting support from local mothers (mentors) for pregnant women or recent mothers identified as at risk of, or experiencing intimate partner violence. Eligible women from the disadvantaged areas of metropolitan north-west Melbourne, Victoria were referred to the study by their family doctors (GPs) or maternal and child health (MCH) nurses during 2006/7 [21].

In theorising what form of social support women vulnerable to violence might value, we drew on current evidence that social support could enhance the mental wellbeing of abused women, irrespective of the level of violence experienced [22]. We also drew from evaluation of three forms of social support previously offered to women experiencing violence *viz*, post-refuge advocacy [23] home-visiting nurses [24,25] and community mentors in antenatal clinics [26]. We sought women whose strongest quality was their capacity to provide empathy and non-judgemental support [27]. Among women experiencing abuse, such qualities are also known to be highly valued in clinicians [28].

Mentors were selected from local mothers with the capacity to provide:

■ a listening, caring ear;

■ non-judgemental support and friendship;

■ assistance with safety strategies for women and their children, including supporting women to access family violence services; and

■ information about, and assistance to access, other relevant local support and services where needed (e.g. legal and court systems, education choices for themselves or their children, language or immigration services).

This model combined forms of social support which Brown (cited in Oakley) distinguished as empathy, nurturance, validation and encouragement, with information and instrumental help [29]. Through enhancing local capacity for providing social support, MOSAIC hoped more broadly to:

■ build sustainability through developing skills, capacity and networks among mentors, women and local services;

■ provide innovative models of good practice for local GPs and MCH nurses – together with stronger networks between primary care providers and community based family violence services, which could enhance positive community engagement in support of very vulnerable families; and

■ provide opportunities for growth and leadership for the mentor mothers themselves – some women had experienced violence themselves and wished to support women going through this experience; others were refugees or immigrants wishing to help their communities and build their skills for later employment in the community.

The MOSAIC training and mentoring experience offered a pathway to further employment and sustained community engagement for the women involved. By advertising for local mothers in local papers, school newsletters and on ethnic radio, MOSAIC was able to recruit 60 women willing to be mentors, of whom approximately 45 mentors were available at any one time over the two years. In seeking to address the double burden of isolation from abuse and migration status, 12 were Vietnamese bilingual mentors supporting Vietnamese women, demonstrating as unwarranted the concerns initially voiced to the research team that Vietnamese women would not volunteer to provide mentoring support, nor would Vietnamese mothers accept it.

### Identification of women for support

MOSAIC participants were pregnant or recent mothers attending primary care services. Intimate partner violence is estimated to be more prevalent among primary care populations than in the general community [30]. This is due to the increased health burden imposed by intimate partner violence, especially for women in their reproductive years [31]. MOSAIC was therefore located in primary care, in order to increase the options for referral currently available in that sector [32] and to trial the provision of support for a population at considerable risk – pregnant women and those with children aged five or less [31]. The ethical imperative not to neglect abused women identified in the comparison arm also informed a decision to train, support and resource all primary care clinicians in the study. Because of the known barriers to clinicians identifying and caring for women experiencing intimate partner violence [33], MOSAIC implemented a number of carefully prepared strategies to enhance primary care clinicians’ capacity to refer. These included: six hours of active, multi-pronged training in intimate partner violence identification and management, innovative GP intimate partner violence clinical guidelines; referral booklets, specially designed posters for the clinic waiting room; information cards about domestic violence services; options for upskilling sessions throughout the year; and regular newsletters [21].

Despite this considerable investment in clinician preparation, the major challenge faced by the study involved the difficulty clinicians reported identifying and referring women. In response, MOSAIC staff conducted interim process evaluation interviews to identify and subsequently address the barriers to clinician identification and referral. Additional MCH nurse training about issues of concern was delivered in conjunction with team meetings, and feedback about women’s positive experiences of the study was provided to clinicians in the comparison arm with further encouragement for referral; but the problem stubbornly persisted, resulting in insufficient numbers of women referred to power the study adequately [34]. Our experiences in MOSAIC indicate that more sustained and sustainable systemic changes – especially improved integration of primary care with community social services, such as family support programs and family violence services – are required for clinicians to feel confident and able to identify and support women experiencing violence.

### ‘Supporting the supporters’

Following a comprehensive recruitment and screening program documented in a manual [27] MOSAIC coordinators provided five days’ training to support mentors in their task. Values clarification and facilitated discussion about the nature of non-judgemental, lay support assisted the practice of empathy among mentors. Discussion of boundaries and the distinction between mentoring and the role of health and other professionals in referred women’s lives also focused mentors’ attention on the importance of their lay support role. Professional involvement may be a double-edged sword for women experiencing intimate partner violence because of the implications of mandatory assessments of the safety of their children. Several women in the mentor group were survivors of violence themselves and well understood that women’s fear of professional surveillance for child abuse often limits their willingness to disclose partner violence and seek support from health professionals or social workers.

Of the 90 women recruited into the intervention arm, 86 responded to the supplementary survey about mentoring. Included in the feedback women provided was this illustration:

*‘I don’t know how I would have survived mentally without Yvonne and MOSAIC. She is better than a social worker because she was able to spend more time with me when she visited and I always had phone contact when I needed to talk. The age gap was good because it was like I had an older sister because I don’t have any family [in Australia] and I had someone to talk to. Yvonne was my only friend. I have had lots of social workers who have visited me but Yvonne supported me through everything…the kids*, *stuff with my ex*, *the legal stuff with Fred*, *moving to a new home*, *getting furniture together…everything.’*

### Managing closure

Preparation for mentoring for a specified time period (up to one year in MOSAIC) had to be made explicit at the beginning of the mentoring relationship. MOSAIC was a research project and not an ongoing service and the option of up to a year of mentoring was made clear to participants in the informed consent process when women were recruited in the intervention arm of the trial. At the eight month review both mentor and woman were reminded that the exit period was approaching. The woman’s connections in her community were explored, her unmet needs were discussed and goals set for the next four months. The coordinator made additional efforts to talk to the mentor over the final three months to facilitate a satisfactory process of closure. Mentors were encouraged to move from a weekly meeting to once a fortnight to ease the farewell. If mentors wished to continue the friendship and some did, it was made clear that they were able to do so, but outside the MOSAIC program and its funding support. In feedback to the study, captured in mentored women’s evaluation surveys, 65/76 (86%) said they thought the time was ‘about right’. Vietnamese women were among those more likely to prefer a longer period of support.

While the outcomes for both intimate partner abuse and depression were in the hypothesised positive directions, the study could only establish qualified support for the value of mentoring in reducing experiences of violence and enhancing maternal wellbeing, due in large part to the under-powering mentioned above [33]. Box 3 provides a brief summary and references for the key features and findings of MOSAIC.

Women’s feedback emphasised the importance of the non-judgemental and empathic aspect of the mentor role. Seventy-nine per cent said they most valued having someone who always encouraged them, and that they could talk about anything that bothered them (78%). Women said that what they had most gained was that they felt better about themselves (61%), less isolated (56%), and a better parent (56%). Eighty-two per cent said that they would definitely recommend mentoring to others.

*‘She had compassion…I could tell her anything*, *she is very trustworthy…* (*she gave me*) *happiness*, *helped me to have more energy…she would always compliment me…encouraging me…we are very compatible.”*

## Discussion

The majority of perinatal support programs aim to improve *child* health and developmental outcomes, with maternal outcomes, such as improved confidence and self efficacy seen largely as mediating improvements in child health, rather than ends in themselves. In contrast the primary aim in both PRISM and MOSAIC was to improve *maternal* health and wellbeing. This focus on maternal issues informed our choices about how to provide support (lay rather than health professional support) and also our choices of outcome measures (maternal experiences of violence, depression, and overall maternal health and wellbeing). In this, our trials have more in common with some of the labour support interventions and the peer support postnatal interventions described earlier.

Based on feedback from our earlier research [35] and from women participating in PRISM and MOSAIC, we are conscious that fear about the judgements of health professionals often holds women back from disclosing depression and intimate partner violence. The perhaps inevitable tension between factors that compromise maternal health, and the impact that this has on child health and development, presents women with a dilemma. Do they disclose issues such as depression and intimate partner violence, and risk judgements about their capacity to provide appropriate care for their children, or is it safer not to disclose these issues, and endeavour to manage them without support?

PRISM and MOSAIC took different approaches to offering women social support. PRISM was a universal strategy to provide all recent mothers with increased opportunities to meet and make friends after the birth of a baby. MOSAIC developed a pool of local women to provide support, matching them with women identified in primary care contacts as vulnerable to intimate partner violence. In PRISM, the universal approach failed to have an overall impact on women’s friendship and wellbeing outcomes at a population level, despite the positive responses of the women who participated in the befriending opportunities made available in intervention communities. With hindsight, there were major challenges for PRISM in managing to achieve sufficient reach and dose of befriending opportunities. While our previous research had shown that a ‘one size fits all’ approach, such as offering new mothers’ groups to all first-time mothers, was unlikely to meet the social support needs of all women, the befriending offerings in PRISM may also have been insufficiently varied and accessible, perhaps especially for women with complex life circumstances or particular vulnerabilities. Befriending in PRISM was an ambitious undertaking implemented across four metropolitan and four rural communities with diverse populations, sometimes quite geographically dispersed. The failure of the intervention strategies to achieve improvements in social connectedness at a community level, may have been due to insufficient tailoring of befriending strategies to reach the most vulnerable groups of women or that the ‘dose’ of befriending experienced was not sufficient to impact on friendships or on depression.

In contrast, the major challenge for MOSAIC proved to be the problem of clinician identification and referral of women, most particularly, but not only in the comparison arm of the trial. In the mentoring arm, nurses and doctors were overwhelmingly positive about the role of mentors – a frequent comment for example, was that women referred to MOSAIC received a quicker response than when referred to other services – but identifying eligible women who were vulnerable to intimate partner violence, remained challenging even after significant training and support. Targeted social support interventions rely on effective identification of women who might benefit from support and this is more difficult than is often assumed. So, although some evidence was seen in the MOSAIC findings that mentoring impacted positively on the experience of violence and on women’s wellbeing, clinician identification and referral problems ultimately affected the power of the study to determine effectiveness robustly, and these findings await confirmation in further studies.

Support provision in both PRISM and MOSAIC valued lay or peer support. While training and ongoing support of mentors was integral to the MOSAIC model, this training was not designed to ‘professionalise’ the women involved, but rather to emphasise the importance in the mentor role of empathy, non-judgemental attitudes and encouragement. The purpose was to provide something different to women from what health or other professionals provide, especially as Victorian mothers are known to have significant contact already with primary health care professionals in the first year after birth. This is rather unlike their counterparts in the US, where primary healthcare remains under-developed, accounting perhaps for the adoption there of nurse home visiting programs to enhance child development. In addition, women experiencing partner violence in Australia also have access to support from other professionals, such as social workers and child protection or family support staff.

The comparative effectiveness of lay versus professional ‘social’ support for mothers is to date rather under-researched. There are major challenges in comparing the diversity both of program types and the range of goals they aspire to, and studies undertaken in the past – particularly in the context of home visiting programs in the US – have sparked debate both about the nature of support being provided and about the definitions of lay (or para-professional) and professional support providers [36-39]. There are at least two key questions here. First, do lay supporters offer something different (better, or more highly valued) than professionals, usually nurses? And second, what are the essential components of support that actively promote health and wellbeing? Can these be offered in both professional and non-professional contexts?

Macdonald *et al* describe common rationales behind professional and lay approaches thus: “Some programmes use professionals as home visitors (on the grounds that parents value ‘expert’ and ‘confidential’ advice and support), whilst others use specially trained and supervised lay visitors (on the grounds that those who have ‘been there’ are better able to engage those who are experiencing difficulty)” [40]. To date, these assumptions underlying lay and professional approaches have not been adequately tested in terms of relative effectiveness. Moreover there is as yet little research identifying the ‘active ingredients’ that might differentiate effective versus ineffective social support and which might also help answer the second key question posed above. There may be lessons to be learned here from recent work on ‘relationship-centred care’, a framework which describes a values foundation for health professionals based upon “meaningful *relationships* in health care, not just on technically appropriate transactions with in these relationships” [41]. Such relationship-centered care is based on respect, recognition of the personhood of the participants (the care provider and the recipient), the importance of ‘knowing’ the person receiving care and their beliefs and values seems to resonate with what women say they value in social support interventions.

One issue arising in this debate focuses specifically on the role of education in the context of providing support for recent mothers. Compared with nurses providing support, lay women trained to provide what had traditionally been a nurse delivered home-visiting program in the US reported feeling uncomfortable with the ‘teaching’ part of the program, commenting that it was ‘patronising’ to mothers [38], and therefore possibly counter-productive in terms of the provision of support. Regardless of whether professional or lay support is offered, the relationship women have with a home visitor and how respectful and non-judgemental the visitor is towards women and their families is likely to be crucially important here, something confirmed in a pilot evaluation of the South Australian nurse home visiting program with Aboriginal families [17]. Other issues raised in the debate concern the amount of training and levels of previous education of lay supporters and the effects this may have on the effectiveness of the support provided. Unfortunately, there has been insufficient rigorous evaluation of these issues, and overall the effectiveness of home-visiting support for new parents remains inconclusive, despite much advocacy for this approach in the US, the UK and in Australia. The findings of a future review (arising from a recently registered Cochrane Review protocol [39]) which will synthesise outcomes from home visiting trials with recent mothers may shed more light on the state of knowledge in this field.

In contrast, the findings of the Cochrane review of continuous support for women in labour are more uniformly in favour of lay supporters (partners, female relatives, ‘doulas’) for achieving improvements in birth outcomes, such as shorter labours, higher rates of normal birth, lower rates of analgesia and greater satisfaction with the birth experience, compared with continuous support in childbirth from a member of the hospital staff [9]. The systematic review of telephone peer support in a range of perinatal contexts by Dennis *et al* found evidence of effectiveness for certain outcomes (breastfeeding duration, smoking relapse, postnatal depression), though not others (preterm birth, smoking cessation) [12]. Lay social support programs do appear to improve wellbeing and some health outcomes for recent mothers, and are deserving of further implementation research.

Questions regarding support duration, appropriate closure when support ceases, and the flow-on effects for women in establishing their own networks of friends for ongoing support, are all areas where research is lacking and future research is also warranted. The challenges of providing appropriate support to mothers in these contexts is partly why in PRISM we opted for attempting to increase women’s ‘opportunities to make friends’ rather than offering women a time-limited peer supporter, or mentor. If, in the context of community-supported befriending opportunities, women had been better able to establish friendships at this time of vulnerability to isolation and lack of support after the birth of a baby, then issues of duration of support and closure would not arise. That PRISM failed to be successful in this more naturalistic approach to reducing isolation and improving maternal wellbeing only underlines the importance of further research on some of these challenges for successfully implementing lay support programs for mothers of new babies. It may also mean that targeted approaches, despite the difficulties of identifying the women in most need of support – such as we encountered in MOSAIC – are those which should be pursued. In addition, studies are sorely needed which aim to test approaches that integrate a focus on maternal health and well-being (caring for mothers) with strategies targeting child health and developmental outcomes, thus developing interventions which name and accommodate the perhaps inevitable tensions in achieving improvements in both.

Finally, it must not be forgotten that the health and wellbeing outcomes of mothers and of children are profoundly influenced by the conditions in which women’s daily lives are lived. As we have seen, support interventions with mothers have often focused on support to improve medical and child health development outcomes, yet it is by addressing the broader social and structural determinants of health – as so forcefully demonstrated in both the recent UK *Fair Society*, *Fair Lives* Report [42] and the Report of the WHO Commission on Social Determinants of Health [43] – and not by the influence of health service activity, that the most profound impacts on women’s and children’s health and wellbeing are likely to be achieved. The power of social connection and support to improve health can only be fully realised in a more just and equitable society.

## Competing interests

The authors declare they have no competing interests.

## Author’s contributions

RS conceived the paper and drafted the section on PRISM with input from SB. AT drafted the section on MOSAIC with input from RS.

RS drafted the Introduction and the Discussion with input from AT and SB. All authors reviewed and revised the final manuscript.

## Supplementary Material

Additional file 1 Click here for file

Additional file 2 Click here for file

Additional file 3 Click here for file
