# Supplementary material for: The power of social connection and support in improving health: lessons from social support interventions with childbearing women
Source: BMC Public Health. 2011 Nov 25;11(Suppl 5):S4. doi: 10.1186/1471-2458-11-S5-S4 (PMC3247027; doi:10.1186/1471-2458-11-S5-S4)
Supplement: Additional file 1 [file 1471-2458-11-S5-S4-S1.docx]

Additional file 1

**Befriending as enacted in PRISM**

**‘Mothers walking together’ -‘Pram walks’ - ‘Walk and talk’ -‘Mothers on the Move’**

Setting up regular times and places to meet for mothers to take part in informal walks together, in twos and threes, (or sometimes larger trains of mothers and prams!) became a popular befriending strategy in several areas. Walks were often facilitated by a local mother/contact person, started at the maternal and child health centre, neighbourhood house or community health centre, and often finished with a cuppa together. When it rained, sometimes the cuppa became the focus!

**PRISM Cafés**

In conjunction with the PRISM voucher scheme in most areas, local cafes and bakeries also offered a special time each week when they invited mothers (and their babies, prams and toys) to join other mothers to meet and talk over morning or afternoon tea. These times were advertised in the PRISM locality guides given to all mothers after they had their baby, and in the local PRISM newsletters for mothers.

**Mothers’ drop-in times**

Advertised times for mothers to ‘drop-in’ for a chat and coffee occurred in a variety of locations: at MCH Centres, neighbourhood houses, churches, community health centres and specialised parenting centres. Often these worked best initially when someone was there to facilitate by getting the kettle on, or talk with the first person to arrive.

**Maternal and child health nurses’ one-to-one befriending initiatives**

Putting mothers in touch with other mothers on a one-to-one basis is something maternal and child health nurses have done for many years: sometimes it might be mothers who live in the same street, but have never met; perhaps for mothers with similar language backgrounds who might find it hard to meet people if their English skills are poor; at other times nurses have facilitated mothers meeting if they both have a child with a similar health problem or disability; or if they have just moved to the areas and are feeling lonely. All these things also happened in PRISM areas, though perhaps these one-to-one offers of befriending occurred with a heightened sense of the potential value of putting mothers in touch with each other as a universal strategy, rather than one targeted to mothers seen as ‘in need’. Some maternal and child health nurses took the initiative to schedule mothers’ appointment times one after the other, or to organise a time with two mothers to meet at the Centre.

**‘Babes in Arms’ – ‘Cry baby’-‘Nursery time’ – baby friendly cinema sessions**

At the time PRISM commenced in local communities in 1999, a small number of metropolitan cinemas had initiated film sessions during the day, designed for mothers (and fathers) who wanted to get out and see a film with their babies in tow. In fact none of the PRISM intervention areas had such sessions occurring locally at the start of PRISM, so the idea of encouraging the local cinema to initiate them as a befriending strategy for mothers as part of PRISM was eagerly taken up by CDOs. Cinemas sometimes also offered mothers a free voucher to attend a ‘babes-in-arms’ session via the PRISM voucher scheme.

**Mothers’ picnics - beach club - outings**

In a number of PRISM communities the idea of mothers getting together to enjoy local physical attractions – the beach, a local park or reserve, a local historical attraction or the town lake – gave raise to mothers’ picnics, barbecues, a ‘beach club’ and outings for mothers and babies. These were very informal affairs, sometimes occurring on a regular basis, once a week or once a month, and sometimes as special events or outings. Outdoor activities being rather weather dependent, some of these were definitely strategies for the milder months!

**Mothers’ Expos – Mothers’ ‘pampering’ days - Workshops for mothers**

In tune with PRISM messages about mothers being supported to care for their own health and to have time out to do enjoyable things for themselves, as well as have opportunities to meet others, several areas organised Mothers’ Expos or ‘pampering’ days, where women were invited to come along and find out about local services, enjoy a massage, or take part in a relaxation workshop or other activities. Babies were welcomed and childcare was also usually available, depending on mothers’ wishes.

**Encouragement of joint PRISM voucher use: something enjoyable with another mother**

One way in which maternal and child health nurses promoted opportunities for mothers to get to know one another was by promoting ‘joint’ voucher use. That is, nurses might suggest to mothers in a first time mothers’ group that they use one of their PRISM vouchers together –go to a babes-in-arms cinema session; do a discounted aqua class at the local swimming pool with another member or members of the group, or use their PRISM café vouchers.

**Library mother and baby sessions and mothers reading together**

Libraries are often places full of mothers and children. Some local libraries started to offer not just children’s story times, but also scheduled mother and baby sessions with songs and nursery rhymes for the babies and a time to chat over a cup of tea afterwards for the mothers. Other sessions involved talks on topics like: relaxation for mothers or myths about parenting. In one PRISM area, promoting mothers reading together was seen as a potential befriending strategy, with the idea of establishing book groups for interested mothers.

**Sporting and physical activities for mothers**

A whole range of befriending opportunities developed around sporting and physical activities designed specifically to meet mothers’ needs in relation to caring for babies and small children. These included: social tennis for mothers, mums’ skate mornings, postnatal exercise classes, indoor bowling, swimming and aqua classes for mothers and babies and tae boxing.

**Mothers’ home mentor scheme**

Mostly, formal befriending schemes were not established in PRISM. In one rural area however, the development of a Home mentor program for mothers was explored with the local community health service, building on a model developed previously for people with psychiatric disabilities. The idea was to train volunteers to provide recognition, a listening ear and nurturing practical support to local mothers experiencing difficulties. The model for the program was developed and funding sought for a program co-ordinator, post-PRISM.

**Internet and computer training for mothers**

Something popular in one rural community was internet training for mothers: mothers online. Getting together for the local training meant meeting other mothers and then networking and chatting online proved positive for mothers in areas ‘out of town’. Another area organised computer training for mothers involved in production of the local mothers’ newsletter.

**Mothers’ involvement in PRISM and advocacy for mothers**

Involvement of recent mothers in all local PRISM steering committees and working groups was an important befriending strategy in itself, something that involved mothers frequently commented on during the project. Coming together to work on PRISM newsletters, to facilitate activities for other mothers or to lobby council for fenced playgrounds and baby change facilities, meant friendships grew in the process. Active mothers’ advocacy groups -‘Mothers in Action’ also formed in some areas.
