# Supplementary material for: The power of social connection and support in improving health: lessons from social support interventions with childbearing women
Source: BMC Public Health. 2011 Nov 25;11(Suppl 5):S4. doi: 10.1186/1471-2458-11-S5-S4 (PMC3247027; doi:10.1186/1471-2458-11-S5-S4)
Supplement: Additional file 2 [file 1471-2458-11-S5-S4-S2.docx]

Additional file 2

# PRISM: key features and findings

PRISM – Program of Resources, Information and Support for Mothers – was a primary care and community-based cluster-randomised trial in sixteen municipalities in Victoria, Australia (1999-2003), which aimed to reduce depression in mothers and improve their physical health following the birth of a baby.

PRISM implemented a range of primary care and community-based strategies in intervention communities, including:

- maternal health and communication skills training for primary care providers,
- information resources for women,
- community development activities to mobilise support for mothers; and
- befriending opportunities to promote support for mothers and decrease isolation.

The main outcome measures, collected by postal surveys of all women who had given birth in participating municipalities (response: 11,305/18,555, 60.8%), included the Edinburgh Postnatal Depression Scale (EPDS) and the SF-36 general health status measure. Analysis was by intention to treat, adjusting for the randomisation by cluster.

At six months after birth, the key findings showed that the PRISM intervention strategies had made no demonstrable impact on:

- women’s depression scores or their overall physical or mental health status;
- women’s opportunities to make friends following the birth of a baby;
- responsiveness to women’s emotional and physical health issues by maternal and child health nurse and general practitioners; or
- mother- and baby-friendliness ratings of local communities.

**PRISM published protocol:**

Lumley J, Small R, Brown S, Watson L, Gunn J, Mitchell C, Dawson W: **PRISM (Program of Resources, Information and Support for Mothers) Protocol for a community-randomised trial [ISRCTN03464021].** *BMC Public Health* 2003; 3:36.

**PRISM primary outcomes paper:**

Lumley J, Watson L, Small R, Brown S, Mitchell C, Gunn J: **PRISM** **(Program of Resources Information and Support for Mothers): a community randomised trial to reduce depression and improve women's physical health six months after birth [ISRCTNO3464021].** *BMC Public Health* 2006; 6:37.
