# Supplementary material for: The power of social connection and support in improving health: lessons from social support interventions with childbearing women
Source: BMC Public Health. 2011 Nov 25;11(Suppl 5):S4. doi: 10.1186/1471-2458-11-S5-S4 (PMC3247027; doi:10.1186/1471-2458-11-S5-S4)
Supplement: Additional file 3 [file 1471-2458-11-S5-S4-S3.docx]

Additional file 3

# MOSAIC: key features and findings

MOSAIC (MOtherS’ Advocates In the Community) was a pragmatic cluster randomised trial based in Victorian primary care (25 general practice and 81 maternal and child health nurse) clinics. The trial tested whether up to one year’s support from a non-professional mentor mother trained to provide domestic violence advocacy, nonjudgmental befriending, self care and parenting support reduced intimate partner violence (IPV) and depression among pregnant or recent mothers at risk of or experiencing IPV.

The main outcome measures were collected by survey and self completed in the home or other safe place, and were delivered and taken away by research staff. These included: IPV (measured by the Composite Abuse Scale (CAS); the Edinburgh Postnatal Depression Scale (EPDS); the SF-36 general health status measure; the Parenting Stress Index (PSI-SF); and the Medical Outcomes Social Support Scale.

At the end of the twelve month intervention period, the trend was strongly in support of the study’s hypotheses, but only one finding was conventionally significant. The key findings demonstrated the following:

- That on average, there was significantly less IPV and fewer experiencing IPV among mentored women than among the comparison group
- That there were fewer depressed women among those mentored compared with those in the comparison group
- That there was improved physical and mental health in the mentored group but no difference in parenting stress between the groups
- That more mentored women had returned to education and training
- That women highly valued the mentor support in their feedback

However, these findings require replication as the study was underpowered due to insufficient referrals from primary care providers.

**MOSAIC published protocol:**

Taft AJ, Small R, Hegarty KL, Lumley J, Watson LF, Gold L: **MOSAIC (MOthers' Advocates In the Community): protocol and sample description of a cluster randomised trial of mentor mother support to reduce intimate partner violence among pregnant or recent mothers.** *BMC Public Health* 2009; 9:159

**MOSAIC primary outcome paper:**

Taft AJ**,** Small R, Hegarty K, Watson L, Lumley J: **Mothers’ AdvocateS In the Community (MOSAIC) – non-professional support to reduce intimate partner violence and depression in mothers: a cluster randomised trial in primary care.** *BMC Public Health* 2011; 11:178
